# One, No One, and One Hundred Thousand: T Regulatory Cells' Multiple Identities in Neuroimmunity

**DOI:** 10.3389/fimmu.2019.02947

**Published:** 2019-12-20

**Authors:** Manolo Sambucci, Francesca Gargano, Gisella Guerrera, Luca Battistini, Giovanna Borsellino

**Affiliations:** Neuroimmunology Unit, Santa Lucia Foundation IRCCS, Rome, Italy

**Keywords:** neuroimmunity, multiple sclerosis, Treg-regulatory T cell, FoxP3, Treg heterogeneity, immune regulation

## Abstract

As the Nobel laureate Luigi Pirandello wrote in his novels, identities can be evanescent. Although a quarter of a century has passed since regulatory T cells (Treg) were first described, new studies continue to reveal surprising and contradictory features of this lymphocyte subset. Treg cells are the core of the immunological workforce engaged in the restraint of autoimmune or inflammatory reactions, and their characterization has revealed substantial heterogeneity and complexity in the phenotype and gene expression profiles, proving them to be a most versatile and adaptive cell type, as exemplified by their plasticity in fine-tuning immune responses. Defects in Treg function are associated with several autoimmune diseases, including multiple sclerosis, which is caused by an inappropriate immune reaction toward brain components; conversely, the beneficial effects of immunomodulating therapies on disease progression have been shown to partly act upon the biology of these cells. Both in animals and in humans the pool of circulating Treg cells is a mixture of natural (nTregs) and peripherally-induced Treg (pTregs). Particularly in humans, circulating Treg cells can be phenotypically subdivided into different subpopulations, which so far are not well-characterized, particularly in the context of autoimmunity. Recently, Treg cells have been rediscovered as mediators of tissue healing, and have also shown to be involved in organ homeostasis. Moreover, stability of the Treg lineage has recently been addressed by several conflicting reports, and immune-suppressive abilities of these cells have been shown to be dynamically regulated, particularly in inflammatory conditions, adding further levels of complexity to the study of this cell subset. Finally, Treg cells exert their suppressive function through different mechanisms, some of which—such as their ectoenzymatic activity—are particularly relevant in CNS autoimmunity. Here, we will review the phenotypically and functionally discernible Treg cell subpopulations in health and in multiple sclerosis, touching also upon the effects on this cell type of immunomodulatory drugs used for the treatment of this disease.

## Introduction

The immune system is called to respond to the environment at each beat of the heart, at each breath, and at each breaching of the organism's surface. In a healthy organism, should an exogenous and potentially dangerous entity gain access, the immediate response of the immune system's defense mechanisms is activated, and clearance of the pathogen is achieved, millions of times a day (1). In addition, immune cells interact continuously with the trillions of bacteria, fungi, and viruses which inhabit, peacefully, the intestines. The interactions with the intestinal microbiota are the second process, after thymic selection, that shapes the immune system. This notion has become clearer in recent years with the knowledge that dysbiosis, an alteration of the physiological composition of the microbial community, underlies the generation of an inflammatory environment which may raise the level of basal immune cell activation thus predisposing to the initiation of inappropriate and uncontrolled immune responses, leading to chronic inflammation and disease (2).

Normally, immune reactions are kept in check by multiple mechanisms which control the magnitude of the response, so escalation to overt inflammation and tissue damage are avoided. However, additional stimuli which may activate immune T cells come also from within, since all the organism's tissues are also highly immunogenic, as exemplified by the immediate rejection of transplants between incompatible individuals. Conveniently, efficient mechanisms of selection during T cell development in the thymus are in place for purging autoreactive cells from the repertoire, and clonal deletion eliminates most of them (3, 4). Inevitably, though, some autoreactive T cells escape this negative selection and circulate in the periphery, and indeed they have been shown to be present at similar frequencies both in healthy individuals and in those afflicted by autoimmune diseases (5–7). But autoimmunity leading to pathology represents the exception rather than the rule, so clearly these autoreactive cells are kept in check in the periphery. Several immune mechanisms exist which mediate restraint of autoreactivity (8), and among these are FoxP3^+^ T regulatory (Treg) cells, whose features and roles in the aberrant immune response which underlies multiple sclerosis (MS) will be discussed.

## Treg Cells in Health

### The FoxP3 Transcription Factor

Treg cells were initially described as a CD4^+^CD25^high^ T cell population whose depletion is associated with the development of severe multi-organ autoimmunity, and were defined by the expression of the transcription factor forkhead box P3 (FoxP3) (9–11).

FoxP3 is capable of binding the astounding number of 2,800 genomic sites, and interacts with more than 360 factors to form a large protein complex (12–15). Four evolutionary conserved intronic cis-elements in the FoxP3 locus regulate the *foxp3* gene expression: conserved non-coding sequence (CNS) 3 is indispensable for the initiation of FoxP3 transcription through the recruitment of c-Rel; CNS2 enables the stable expression of FoxP3 in actively proliferating Tregs, and CNS1 is key for the extrathymic induction of Tregs in the periphery, and contains binding sites for TGF-β (16). Last to be discovered, but actually the pioneering element, is CNS0, crucial for the establishment of the earliest epigenetic modification controlling FoxP3 expression (17). Interestingly, methylation at these crucial sites is affected by cytokine signaling and by environmental cues, thus it is possible that the inflammation which accompanies autoimmunity may have an impact on this “basic” epigenetic regulation and stability of FoxP3 (18).

Stable FoxP3 expression also relies on epigenetic modifications of the Treg-specific demethylated region (TDSR), a non-coding region in the first intron of the *foxP3* gene locus (19, 20), and this has become the marker of “true” Treg cells, allowing discrimination from activated CD4^+^CD25^+^FoxP3^+^ cells. The presence of DNA hypomethylation at Treg signature genes contributes to the maintenance of lineage stability, and does not occur in activated cells which transiently express FoxP3 and which lack suppressive abilities (21).

Additionally, similar to most transcription factors, FoxP3's function can be modulated by post-translational modifications (such as ubiquitination, acetylation, and phosphorylation), which couple extracellular cues to adjustments of transcriptional programmes [for a review see (22, 23)].

In humans, several splicing variants of FoxP3 have been described (24). The splicing variant containing exon 2 (FoxP3-E2) is the better equipped for interaction with RORα and RORγt, two transcription factors involved in Th17 specification (25, 26). Metabolic and cytokinic factors determine alternative splicing, and we and others have shown that, in patients with MS, Treg cells express reduced levels of FoxP3-E2 and are thus deprived of an auxiliary level of regulation (27, 28).

The Treg phenotype needs to be “locked in” and stabilized, since these cells are self-reactive and their conversion into conventional effector cells would unleash a dangerous army of autoimmune effectors (29). So how do Treg cells resist acquisition of conventional T (Tconv) cell properties, in inflammatory environments? FoxP3 prevents the expression of genes encoding effector cytokines by acting as a repressor or an activator and through the physical interaction with other transcription factors (30, 31). These aspects are discussed below.

## Treg Cell Development

*In vivo*, most Treg cells develop in the thymus (tTreg) during positive selection as an alternative to conventional CD4^+^ T cells, but they also may be generated in the periphery (pTreg) through extra-thymic conversion of conventional T cells.

In the thymus, T cells recognizing self-antigens are deleted, except for a small fraction of cells, which is pushed down the path of Treg differentiation, largely due to the avidity of the MHC-peptide interaction that their TCR establishes with cortical thymocytes (32). Specifically, diversion in the Treg lineage occurs when the affinity of the TCR for self-peptides ligands falls between the range of positive selection of conventional T cells and negative selection of T cells carrying TCRs with high affinity. Thus, strong TCR signaling is the prerequisite for Treg development (33). In addition to TCR signaling, other factors, such as signaling through CD28 (34), the presence of IL-2 (35) and of TGF-β (36) are required for Treg differentiation. Following this first step of TCR-dependent maturation, CD25 upregulation occurs, making tTreg precursors responsive to the IL-2 signals, which are transduced by STAT5 and are necessary for the induction of the master regulator FoxP3 (37). Interestingly, in the thymus there is limited availability of IL-2, which may be further reduced following sequestration by mature activated Treg cells recirculating to the thymus and limiting further generation of Treg cells (38). Acquisition of FoxP3 expression by thymocytes coincides with the emergence of phenotypic and functional features of mature peripheral Tregs, including inability to produce IL-2 and emergence of suppressive abilities. Genome-wide analyses of FoxP3 have shown that it binds to ~700 genes and miRNAs involved in multiple networks, acting both as a repressor and an activator (14, 39); FoxP3 also forms multiprotein complexes with over 360 proteins and acts in cooperation with FoxP1, another member of the FoxP family, in the regulation of gene expression (40, 41).

However, FoxP3 expression alone is not sufficient to establish the full Treg cell phenotype, as indicated by the fact that ectopic expression of FoxP3 fails to induce a large proportion of Treg cell signature genes, and T cell activation in humans transiently induces high levels of FoxP3 but not suppressive abilities (42–44). Thus, in addition to FoxP3 expression, a second process is necessary for the full and stable development of the Treg lineage, and that is the establishment of a specific epigenetic signature (45–47). Tregs in fact exhibit a specific DNA hypomethylation pattern (48) particularly at Treg cell signature genes, such as *FoxP3, CTLA4, IL2ra, and Ikfz2*. Chromatin accessibility of the genes defining Treg cell identity is facilitated by activation of super-enhancers (SE) in Treg precursors, and the genomic organizer Satb1 has a pioneering effect through “preconditioning” of the chromatin landscape and binding to Treg-SE at the early stages of thymic Treg development (17). Interestingly, the *SATB1* locus contains single nucleotide polymorphisms (SNPs) associated with MS (18), suggesting that alterations in the initial events that lead to the generation of Tregs may contribute to genetic susceptibility to immune dysregulation and to disease development. The presence of DNA hypomethylation at Treg signature genes contributes to the maintenance of lineage stability, and does not occur in activated cells which transiently express FoxP3 and which lack suppressive abilities (21).

In addition to Tregs generated in the thymus, peripheral conversion of Treg cells occurs in some organs, such as the colon, where pTregs emerge following encounter with commensal bacteria and their metabolites (49–53), and in the placenta, where they mitigate maternal reactivity to the fetus (54, 55). FoxP3 induction is dependent on the FoxP3 enhancer CNS1, and selective ablation of pTregs in CNS^−/−^ mice induces spontaneous development of pronounced Th2-type inflammation in the gastrointestinal tract and lungs, with concomitant alterations in the composition of the gut microbiota (16, 56).

It has long been known that thymectomy before day 3 after birth induces severe autoimmunity, indicating that pTregs alone are insufficient for the maintenance of cell tolerance (11). Current thinking is that pTregs suppress inflammation at mucosal barriers where they are key contributors to the maintenance of tolerance to a vast array of foreign antigens derived from the microbiota and from the diet, while tTregs control immune responses to self antigens (56, 57). A healthy immune system results from the balanced interaction between the antigenic blast present at barrier surfaces and the active suppression provided by Treg cells. The distinction between pTregs and tTreg is only one of several that can segregate these cells in different subsets, in a graded scale of differentiation and function that we describe below.

### Treg Cell Heterogeneity

In humans, all circulating CD4^+^FoxP3^+^ tTreg cells are characterized by high levels of CD25 and low CD127. However, they comprise different phenotypic subsets, which can be separated based on expression of CD45RA and CD25 in naïve (CD45RA^+^CD25^+^FoxP3^+^) and effector (CD45RA^−^CD25^+^FoxP3^+^) cells; a third subset, which expresses low levels of FoxP3, contains a mixed population of recently activated Tconv cells which do not have suppressive functions and produce cytokines at high levels (44). Naïve Tregs are likely recent thymic emigrants, and accordingly a high fraction express CD31 (58); following activation they differentiate in effector Tregs (44). Effector Tregs express high levels of suppressive molecules, such as CTLA4. A recent study investigating the proteomics and transcriptomics of these subsets has defined both a common Treg and an effector Treg signature, which is maintained even after stimulation and expansion *in vitro*. The main features of the Treg signature are the stability of FoxP3 expression and the inability to produce effector cytokines, the latter characteristic being achieved by the low abundance of transcription factors, such as STAT4, a major activator of IFNγ transcription (59).

The effector and naïve phenotypes are associated with differential expression of other molecules, such as CD39, CCR6, HLA-DR, GITR, LAG3 (60, 61), which are involved in the suppressive function and migratory ability of Tregs, and which may be expressed at different times during the cells' life.

ICOS expression defines two other Treg subsets, and is associated with ability to secrete IL-10 together with TGF-β. FoxP3^+^ cells receive a different imprinting during thymic development, depending on the presence or absence of costimulation through ICOS and on the thymic antigen presenting cell they interact with (62).

A specialized subset of Treg cells expresses TIGIT, a coinhibitory molecule, and gene profiling of TIGIT^+^ and TIGIT^−^ Treg cells indicate that TIGIT^+^ cells have the attributes of a highly suppressive population specialized in suppression of Th1 and Th17 responses, with increased levels of expression of CTLA4, PD-1, Tim-3, and in humans also of CD39 and CCR6 (63, 64).

Thus, Treg cells can be defined on the basis of the expression of surface markers which identify distinct stages of differentiation and which denote the suppressive competence of each subset.

## Treg Cell Plasticity

Conventional T cells differentiate into distinct types of effector cells when exposed to particular combinations of cytokines produced by innate immune cells following infection with specific classes of pathogens. The expression of lineage-specific transcription factors and epigenetic modifications underlies the full differentiation of distinct T cell subsets which can optimally deal with the invading pathogens (65). Several Th subsets have been identified to date, and following the initial discovery of the Th1 and Th2 subsets, Th9, Th17, Th22, and Tr1 subsets have been subsequently described. The effector function of these Th subsets mainly consists in the specialized secretion of the most appropriate set of cytokines for each category of challenge, so Th1 cells produce mainly IFNγ in their fight against intracellular bacteria, Th2 cells secrete IL-4, IL-15, and IL-13 to contrast helminths and parasites, and IL-17 produced by Th17 cells protects from fungal infections.

**Figure 1:**
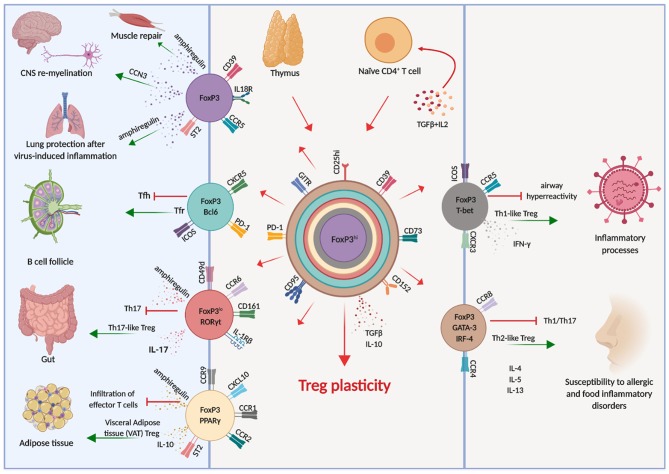
Treg cell plasticity. Treg cells have the potential to acquire expression of the molecular machinery which enables them to adapt to different environments, shadowing effector T cells and tissue-resident cells. This potential is schematically illustrated by the different colors, each corresponding to distinct cellular localization. Arrows indicate the known pathways of functional differentiation of Tregs and the tissues and organs where they can localize.

It soon became clear, both in the human and in the murine systems, that also Treg cells have a considerable degree of plasticity, exemplified by the co-expression of multiple transcription factors typical of other CD4 T cell lineages (66). This allows Treg cells to adapt to their location and to be equipped with the surface receptors, effector molecules, and metabolic assets matching those expressed by activated effector cells (Figure 1). Indeed, Tregs are able to position themselves in all tissues, wherever immune cells are activated and need to be regulated. Through the vast arrays of chemokine receptors and adhesion molecules they can swarm to non-lymphoid tissues just as conventional T cells do (67), showing that also Tregs have an inherent flexibility which enables adaptation to changing environments and focused immune regulation.

For instance, in response to IFNγ Treg cells express T-bet, which in turn induces expression of CXCR3 and Treg cell accumulation at sites of Th1 mediated inflammation (68), and loss of T-bet-expressing Treg cells causes severe Th1 autoimmunity (69). Relevant to immune reactions occurring in the brain, the CXCR3 chemokine receptor guides cells who express it toward CXCL9, CXCL10, and CXCL11, which are released by both neural and immune cells. Once T cells, both conventional and regulatory, have entered the brain, they are retained by CXCR3 in the perivascular zone rather than spreading in the white matter. This restricts the area of inflammation and favors the interactions between cells, enabling cell contact-dependent mechanisms of immune regulation (70).

Similarly, the microbiota in the intestine induces Treg cells expressing RORγt and STAT3 (71–73), which colocalize with RORγt-expressing Th17 cells. Moreover, high levels of expression of interferon regulatory factor-4 (IRF-4), one of the transcription factors involved in Th2 differentiation, is dependent on FoxP3 expression and is required to efficiently restrain Th2 responses (74). Treg cells can also acquire expression of Bcl6 which induces CXCR5, enabling them to accumulate in germinal centers and to regulate humoral immunity, including B cell affinity maturation and plasma cell differentiation (75).

Although Tregs respond to inflammatory cues and express the master transcriptional factors of Tconv cells, they do not produce the respective cytokines and they maintain stable expression of FoxP3 (56, 76, 77), although it is possible, in conditions of extreme inflammation, that FoxP3 expression is lost and conversion to conventional cytokine-producing cells is reached (78–80). In these cases, “ex-FoxP3” cells acquire the ability to produce IFNγ and are pathogenic in the experimental model of MS, experimental autoimmune encephalomyelitis (EAE) (78).

## Treg Cell Mechanisms of Action

To—literally—gain a picture of Treg's mode of action, a good starting point is the definition of their tissue distribution and, more microscopically, of their positioning relative to the other immune cells during immune responses. tTregs circulate in the periphery and populate peripheral lymph nodes, where at the steady state they constitute 10–15% of the CD4 cell pool (81, 82). Here, Tregs aggregate in clusters with activated autoreactive effector CD4 T cells, which they can effectively “govern” given their close proximity, thus suppressing incipient autoimmunity (83). An interesting study showed that the first responders to the IL-2 produced in the initial phase of the immune responses are STAT5^+^ Treg cells, which are promptly activated and form a “safety net” constraining subsequent activation of conventional T cells (84). Indeed, Treg express very high levels of CD25, which enables them to capture IL-2, thereby depriving conv T cells of the primary cytokine which sustains their proliferation, and inducing apoptosis (85). This ability has earned Treg cells the epithet “IL-2 sink,” although subsequent studies challenge the notion of IL-2 deprivation as a prominent mechanism of suppression, at least *in vivo* (86).

Once they are positioned in close proximity of the cells they need to regulate, Tregs use different molecules to oppose Tconv activation, the most prominent of which is CTLA4, which captures its ligands CD80 and CD86 thus making them unavailable for binding to CD28 on Tconv cells (87). Moreover, CTLA4 has a higher affinity for CD80 and CD86 than does CD28, thus CTLA4^+^ Tregs outcompete CD28-expressing T conv cells for interaction with Dendritic Cells (DCs) (88). Also, CTLA4 induces upregulation of the tryptophan catabolizing enzyme indoleamine2,3-dioxygenase (IDO) by DC, inducing their regulatory phenotype (89). This modulation of antigen presenting cell maturation and function represents a major mechanism used by Treg cells to suppress immune responses.

Endangered or dying cells release potent proinflammatory stimuli, such as ATP, which Treg cells are able to catabolize through the ectoenzymes CD39 and CD73, which degrade ATP to AMP and adenosine (90, 91). Nucleotide catabolism achieves dampening of the immune response through both the removal of an inflammatory molecule (ATP) and the generation of highly immunosuppressive adenosine, which can bind to its receptors expressed on the surface of all immune cells (92, 93). CD39 expression identifies Tregs with an effector-memory phenotype, and these cells are more stable and present increased functionality (94). This pathway is of great interest as in addition to a general downregulation of aspecific inflammation, it also impacts on the generation of pathogenic Th17 cells. ATP is a potent activator of the inflammasome, with consequent release of the proinflammatory cytokine IL1β (95), and it also induces IL-23 release by a subset of DCs (96), two cytokines involved in Th17 differentiation. Moreover, ATP is directly involved in the generation of Th17 cells in the intestine (97), the major site of antigenic challenge and consequent cell activation. Thus, CD39^+^ Tregs cells at sites of inflammation may contrast the generation of pathogenic effectors. Interestingly, CD39 expression can also be induced in Th17 cells conferring immune-suppressive properties (98), and during the resolution of inflammation, Th17 cells can transdifferentiate into Tregs (99), with CD39 expression correlating with the ability to produce IL-10 (100). Thus, Th17 and Treg cells' functions intersect also in this pathway, and in this regard it is interesting to notice that while human Treg cells only express CD73 at minimal levels, if any, Th17 in inflammatory sites have high levels of CD73 on their surface (101); thus, the generation of immune-suppressive adenosine through the concerted action of CD39 and CD73 may occur when Th17 and Treg cells are in close proximity, as happens in sites of immune regulation (83).

Other mechanisms of action include the release of immune-suppressive cytokines, such as TGF-β (102), IL-10 (103), IL-35 (104), and of cytotoxic molecules, such as perforin (105), granzyme (106), and cAMP (107, 108).

Treg cells may also acquire tissue-specific abilities, not limited to immune suppression. Tregs stably express high levels of the enzyme 15-hydroxyprostaglandin dehydrogenase, particularly in the visceral adipose tissue, which enables them to catabolize PGE2 into 15-keto-PGE2, which in turn inhibits the proliferation of CD4^+^ Tconv cells (109). In the muscle, Tregs participate to tissue repair through the secretion of amphiregulin, which facilitates the regeneration of muscle satellite cells (110); similarly, amphiregulin production by Treg cells helps contain the damage to lung tissue following infection (111).

In the CNS, in a mouse model of lysolecithine-induced focal demyelination, Treg cells have been shown promote oligodendrocyte differentiation and remyelination through the release of CCN3, a growth-regulatory protein usually expressed in the developing brain (112). Moreover, ablation of Treg cells during acute spinal cord injury delays tissue remodeling (113).

The skin, another barrier site constantly exposed to exogenous antigens, hosts significant numbers of Treg cells (114), although curiously these are mainly represented by tTregs which colonize this tissue shortly after birth (115). Skin Treg have been shown to facilitate cutaneous wound healing and epithelial stem cell differentiation through expression of Jagged 1 (116), and to regulate fibroblast activation and fibrosis through expression of a skewed transcriptional programme dominated by GATA3 (117).

Thus, Treg cells can adopt several mechanisms for the down modulation of immune responses at both in lymphoid and non-lymphoid tissues, which generally safeguard against pathological and autoimmune reactions.

## Tregs and the Microbiome

In the words of Blaser, the microbiota is at the same time self and non-self, being part of our biology but rapidly changing in response to external stimuli (118). While tolerance to self-antigens is instructed in the thymus, post-thymic education to tolerate foreign antigens occurs in the intestine, which is colonized by trillions of microbes with whom a healthy symbiotic relationship is established. The immune system shapes and preserves the ecology of the microbiota, which in turn tunes and calibrates immune cells, in a continuous homeostatic dialogue (119, 120). Changes in diet, improved sanitation, and mindless use of antibiotics have had a profound impact on the microbiota composition of contemporary populations in western countries, reducing diversity and promoting enrichment with bacterial strains capable of inducing inflammation, mainly through the generation of Th17 cells, but also through decreased induction of suppressive Tregs. These changes in microbiota composition are thought to contribute significantly to the increase in autoimmune and inflammatory diseases observed in recent times (2, 121).

Gut Tregs, which represent a large proportion of mucosal T cells interacting with the commensal microbes, are generated both through the peripheral conversion of Tconv cells in response to the microbiota (49, 51, 52, 56, 73), or following colonization and expansion of tTreg (50, 122–124), thus establishing tolerance, and actually also influencing the composition of the microbiota: Treg cell deficiency is accompanied by gut dysbiosis (125), and impaired pTreg generation leads to perturbations in the composition and metabolic function of intestinal microbiota (126).

Metabolites produced by the commensal bacteria also influence Tregs. For instance, short chain fatty acids (SCFA) including butyric and propionic acid induce the differentiation of Treg cells through H3 acetylation at the CNS3 and CNS1 regions of the FoxP3 gene locus (49, 127). Polysaccharide A (PSA) produced by the commensal *Bacteroides fragilis* prevents intestinal inflammatory disease and induces the generation of IL-10-producing FoxP3^+^ Treg cells (128–130). These expanded Treg populations express CD39, which in addition to its role in catabolizing ATP, also modulates their migratory capacity (131).

The microbiota consists also of organisms that direct pro-inflammatory immune responses and Th17 differentiation. Th17 are essential for the protection against extracellular bacteria and fungi (132–134), but their pathogenic role in inflammatory disease is also well documented. Commensal microbiota are involved in the development of intestinal Th17, with *Candida albicans* playing a prominent role (132) followed by segmented filamentous bacteria (SFB), which promote Th17 differentiation and brain inflammation (135, 136).

**Figure 2:**
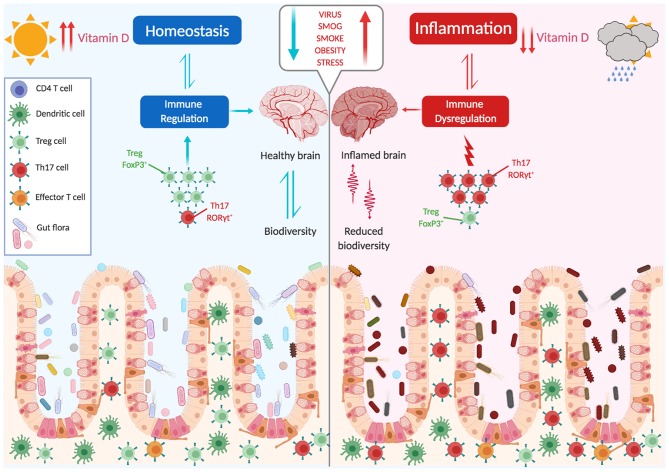
The microbiota shapes immune responses. The balance between proinflammatory and immune suppressive factors (dysbiosis, vit. D, stress, obesity, smoking, infections, diet) is critical for the maintenance of tolerance and avoidance of autoimmunity. Several environmental factors can influence this balance, with a main role being played by the gut microbiota.

The picture that emerges is that the composition of a healthy microbiome is not accidental, and was established after millennia of coevolution. The balance between the generation of proinflammatory effector cells, which combat potentially pathogenic microbes, and immune-suppressive regulatory T cells, which oppose and downmodulate these responses, is an active process. Changes in this balance, either due to an altered composition of the microbiota or to an intrinsic defect of Tregs, may generate inflammatory responses that target organs distant from the gut, as the brain (Figure 2).

## Neuroimmunology: Multiple Sclerosis

Multiple sclerosis is an inflammatory disease of the central nervous system characterized by recurrent attacks of neurological dysfunction from which early on in the disease course patients gradually recover; in most patients, a phase characterized by progressive clinical worsening then ensues, and permanent disability is established (137–139). Pathologically, MS is characterized by the appearance of demyelinating lesions with extensive inflammatory infiltrates both in the white and in the cortical gray matter, pointing to an involvement of the immune system in disease pathogenesis. Indeed, genome-wide association studies (GWAS) have indicated that virtually all MS-associated genes are involved in immune processes (18, 140, 141), with expression of the HLA-DRB1^*^15:01 allele being the most dominant risk factor. This genetic association argues strongly in favor of the central role of the adaptive immune response in MS, since T cell activation sparks from the interaction of TCRs with HLA molecules.

MS arises for an unfortunate combination of factors and events, and different risk factors have been identified. The genetic component is evidenced by a high concordance rate in monozygotic twins (25–30%), but the fact that in this case the relative risk does not reach 100% points to a role of environmental factors in inducing disease. Low vitamin D levels, cigarette smoking, the presence of environmental toxins, obesity, and infection with EBV virus are the most consistent environmental factors linked to MS (142, 143). In more recent years, the influences of the microbiome in inducing or preventing MS have emerged. Alterations in the composition of the microbiome in contemporary populations is thought to be partly responsible for increases in autoimmune disorders including MS. An interesting paper by Vatanen et al. (144) showed that depending on the composition of the microbiota, LPS derived from different strains has different immunogenicity and may lead to altered immune maturation and predispose to autoimmunity. Indeed, the intestinal microbiome of MS patients shows distinct changes compared to healthy controls, particularly with a significant reduction in biodiversity (145–148). Interestingly, fecal transplants from MS patients in transgenic models of brain autoimmunity precipitate disease (149, 150), further supporting a role of microbial composition in inducing pathological immune responses.

Dysbiosis, with a shift in the microbiota composition toward a proinflammatory asset, also determines alterations in intestinal permeability, granting access of bacterial components and preceding the onset of brain autoimmunity in the experimental model (151, 152).

The CNS is kept separate from the rest of the organism and from the outside environment by several anatomical and cellular layers, in order to protect the resident cells which are particularly vulnerable to immune-mediated damage and unable to regenerate. The meninges surround the brain and contain the cerebrospinal fluid, and are also host to a functional lymphatic system which enables drainage from the CNS to the deep cervical lymph nodes (153, 154). Although it is equipped with a lymphatic system, the brain is an immunologically unique site, and it is shielded from the blood circulation by a specialized vasculature composed by endothelial cells joined together by tight junctions and surrounded by glial podocytes, thus creating a nearly impermeable boundary, the blood brain barrier (BBB) (155), which represents a limiting factor to the entry of metabolites and of immune cells into the CNS. Alterations of the permeability of the BBB clinically correspond to the formation of the typical acute gadolinium-enhancing regions visible by magnetic resonance imaging (MRI) scans. Interestingly, the BBB is vulnerable to changes in the gut microbiota (156), and coloniziation of mice with a single organism, *B. fragilis*, can restore permeability (157).

Activated and memory T cells in the periphery express the adhesion molecules and chemokine receptors which enable them to cross the BBB and to infiltrate the brain tissue (158, 159), and this feature is exemplified by the induction of neuroinflammation in the murine model through the transfer of myelin-specific T cells which upon injection in naïve mice rapidly accumulate in the brain, generating the typical lesions similar to those observed in MS patients. CCR6 expression has been shown to be a requirement for migration in the CNS (160), following a gradient of CCL20 produced by the choroid plexus, which allows entry in the brain ventricles. Once in the tissue, these cells can become reactivated following interaction with resident antigen-presenting cells (APCs), such as microglia or myeloid cells, and can start producing inflammatory cytokines which increase BBB permeability and initiate the cascade of infiltration by pathogenic proinflammatory cells, and thus the typical perivascular infiltrates are formed (161). CD8^+^ T cells also gain access to the CNS, and actually constitute the majority of the T cell infiltrate; through the release of cytotoxic molecules, such as perforin and granzyme they induce demyelination (138, 162, 163), and they have also been shown to produce IL-17 (163). “Special” CD8 T cells also participate to disease: mucosal-associated invariant T (MAIT) cells (164, 165), a subset of innate-like T cells which recognize molecules derived from bacterial metabolism and which usually reside in the gut, where they control the intestinal microbiome. These cells have been found in the infiltrating lesions of MS patients, where they produce IL-17. Also B cells have a pathogenic role in MS (166): the presence of oligoclonal bands is a hallmark of the disease, and ectopic B cell follicles are present in meninges from patients with MS (167, 168). Moreover, the spectacular results obtained in clinical trials using antibodies targeting B cells confirm the role that these cells play in the disease, including their role as APC which may reactivate autoreactive T cells crossing the BBB (169–171).

Together with these pathogenic cells, also Tregs gain access to the CNS.

## Treg Cells in Multiple Sclerosis

The CNS is an excellent organ to study in models of inflammatory autoimmune disease, since physiologically it is free from activated immune cells, such as lymphocytes and macrophages. The induction of an autoimmune reaction directed against CNS components—typically myelin antigens—is a well-established model for human multiple sclerosis, and has provided crucial insight on the immune mechanisms underlying the disease.

The initial studies on Treg cells in MS were those performed in the murine model of brain autoimmunity, where it was discovered that their ablation aggravated disease, while adoptive transfer inhibited it (172, 173). Several studies then addressed the issue of Tregs in MS patients, which showed that Treg cell frequency as a whole, mostly determined by the gross measurement of CD4^+^CD25^high^ circulating cells, was shown to be in the physiological range (174–177), or decreased (178, 179). However, investigations on the functional abilities of these cells with the identification of distinct subsets have revealed impairments at different levels which partly explain the defects in immunoregulation underlying the autoimmune attack in MS (180–182).

It has been shown that the frequency in the peripheral blood of Tregs expressing CD39 is reduced in MS patients, and that this correlates with a lower ability to catabolize ATP (90). Interestingly, CD39^+^ Treg cells are able to efficiently suppress IL-17 production by Th17 cells, contrary to their CD39^−^ counterpart, thus their reduction in MS patients may participate to the ineffective control of Th17-driven inflammation in this disease (183). A recent study has shown that in MS patients experiencing clinical relapses the frequency of CD39^+^ Treg cells was increased (184), possibly in an attempt to counterbalance active inflammation (185). In the murine model, PSA produced by the human intestinal commensal *Bacteroides fragilis*, induces expansion of CD39^+^Treg cells with protective effects on EAE manifestations, and ablation of CD39^+^ abrogates these effects (186).

Treg cells from MS patients have been shown to express lower levels of FoxP3 (187), and a more detailed study later showed that in patients there is prevalent expression of the FoxP3 isoform lacking exon2, making Treg cells less efficient in inhibiting the development of proinflammatory cells (28).

Genetic polymorphisms in Treg-relevant genes may also underlie the development of MS. For example, a high risk of MS is associated with polymorphism in CD25 (188).

Also resident cells of the CNS can influence Treg cell activity. Neurons, for instance, produce TGF-β and might directly affect T cells. One study has shown that neurons participate to the conversion of encephalitogenic CD4 T cells to Treg cells expressing FoxP3 and mediating suppression through CTLA4 (189). Moreover, serotonin, a CNS neurotransmitter, favors the expansion *in vitro* of Treg cells from MS patients while reducing production of IL-17 by Tconv cells, suggesting that it acts as a neuroprotectant in the attempt of resolving inflammation (190).

Adding to all this, another aspect is resistance of Tconv to the suppressive action of Treg cells: effector T cells may “break free” and escape Treg suppression, mainly through accelerated production of IL-6 and STAT-3 signaling, as has been shown to happen in MS patients (191–193).

**Figure 3:**
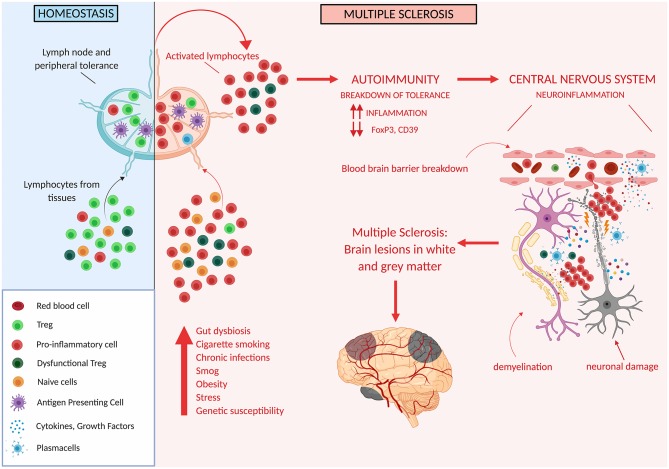
Tregs in multiple sclerosis. Genetic and/or environmental factors may induce hordes of proinflammatory cells and thus may overcome the ability of Treg cells to suppress inflammation. This may unleash autoreactive immune cells which cross the blood brain barrier and target different areas of the CNS ultimately determining the development of white and gray matter lesions.

Thus, Treg impairment at different levels has been described to occur in MS patients, and likely plays an important role in determining altered immune regulation and disease susceptibility (Figure 3).

## Effects of Immunomodulatory Drugs for MS on Tregs

Although there is no cure for MS, 15 drugs are now available for modifying disease course of the relapsing-remitting form (194, 195). Early treatment is recommended in order to achieve the goal of “no evidence of disease activity” (NEDA) (196), and currently no patient is left without therapy. All these treatments target the immune system, and most also exert an effect on Treg cells.

The first injectable drugs approved for use in MS were type I interferons and glatiramer acetate (GA), and they are used as first-line agents to slow disease progression. Type I interferons have a plethora of immunomodulatory functions, in addition to their role in viral interference (197, 198). In MS patients, treatment with this drug enhances regulatory cell function and increases FoxP3 mRNA levels, thus favoring immune suppression (199–201). GA is a synthetic polymer of aminoacids, whose complex mechanism of action are still incompletely understood, and involves both immunomodulatory and neuroprotective effects. Patients treated with GA show increased levels of FoxP3 (202) and of Treg function (203), seemingly through a direct effect on thymic output of naïve Tregs.

Natalizumab is a humanized monoclonal antibody directed against the α4 chain of α4β1 integrin (CD49d), and it interferes with lymphocyte migration in the CNS. It is highly effective in reducing the annual relapse rate (204, 205), although it may predispose to developing opportunistic infections in the CNS. Interestingly, contrary to Tconv cells Tregs express very low levels of CD49d (206) thus their migration to the CNS may not be affected by therapy with natalizumab. Indeed, given the low expression of CD49d by Treg cell, the therapeutic effects of this drug seem to be independent of a direct effect on Tregs (207).

Fingolimod was the first oral drug to be approved for use in MS. It is a S1P_1_ antagonist, thus it prevents T cells from exiting the lymph nodes, with the result of reduced numbers of circulating T cells (208, 209). However, it is now clear that the effects of fingolimod on immune cells go beyond the modulation of their migratory properties. In Treg cells S1P_1_ inhibition leads to increased thymic egress and suppressive function, through blockade of the Akt-mTOR pathway (210). Deletion of S1P_1_ in a mouse model induced development of systemic autoimmunity, and acute S1P_1_ ablation enhanced susceptibility to EAE (211). In the same study it was also shown that in patients undergoing treatment with fingolimod the phenotypic conversion from naïve Treg cells to effector T cells occurs with greater frequency. Fingolimod also affects Treg plasticity by reducing the expression of T-bet and of IFNγ and by enhancing expression of Tim-3, a marker associated with superior suppressor capacity (212). In general, these studies show that Treg function is increased following treatment with fingolimod.

Dimethyl fumarate (DMF) is an oral drug which was initially used to treat psoriasis, and was later approved for use in MS (213). The mechanism of action is still not completely understood, but seems to rely on the activation of the nuclear-related factor 2 (Nrf2) antioxidative response pathways (214). Very few studies have addressed the impact of DMF directly on Treg cells, and besides an increase of FoxP3^+^ cells during the first months of treatment (215), no other effect has been described.

Alemtuzumab is a humanized monoclonal antibody specific for CD52, a protein highly expressed by T and B cells and at lower levels on innate immune cells. Two trials established its safety and efficacy in MS patients (216, 217). Treatment with alemtuzumab induces long-lasting lymphopenia, and the beneficial effect on MS seems to be due on the re-equilibration of the immune system which occurs through depletion and repopulation of lymphocytes, which includes an enrichment in Treg cells (218, 219), and an increase of Treg suppressive function (220).

Cladribine (CdA) is a synthetic deoxyadenosine analog. Like deoxyadenosine, it enters lymphocytes through an efficient transport mechanism. Once in the cell, deoxyadenosine has two potential fates: irreversible deamination by adeonine deaminase (ADA), leading ultimately to the uric acid excretion pathway, or serial phosphorylation by deoxycytidine kinase (DCK) to dATP. Cladribine, on the other hand, is resistant to deamination by ADA because of its chlorinated purine ring structure and thus can only phosphorylated by DCK to its lymphocytotoxic form, 2-chlorodeoxyadenosine triphosphate, which accumulates and is incorporated into DNA. This results in DNA strand breakage and inhibition of DNA synthesis and repair, and to cell death. The selective toxicity of CdA toward both dividing and resting lymphocytes makes the drug useful as an antileukemic or immunosuppressive agent, and indeed it is used in hematologic cancers, such as B cell chronic leukemia and particularly hairy cell leukemia (221, 222). Subsequently, the drug was tested for treatment of MS. The “CLARITY” trial for relapsing remitting MS showed a significant reduction in relapse rate (223) and the drug is now approved for treatment of “rapidly evolving severe” MS (defined as at least two relapses in the previous year and an MRI scan showing new, or bigger, lesions). The beneficial effects of cladribine seem to be achieved through targeted and sustained reduction of circulating T and B lymphocytes, but it is possible that additional immunomodulatory actions participate to its effects (224). MS patients that express low levels of CD39 are likely to respond to cladribine therapy, since they lack the level of immune-regulation provided by the generation of suppressive adenosine, and thus may benefit from pharmacologically induced increases in adenosine.

From this survey of the effects of currently used immunomodulatory drugs it emerges that the most frequently measured effect is an increase in frequency and in suppressive function of Tregs, and although these cells are not the direct target of these therapies, nonetheless the improved immune regulation they provide contributes to altering the progression of the disease.

## Conclusions and Future Directions

The Treg cell population is vastly heterogeneous, as most complex makings of Nature, and comprises an army of cells expressing graded levels of transcription factors and of effector molecules. FoxP3 dominates the machinery of these cells, and through the interactions with both the genes made accessible by epigenetic mechanisms and with the nuclear proteins available for binding, it regulates transcription of the quintessential Treg molecules. Treg differentiation is a plastic and actively maintained state determined by the collective activity of the whole transcriptional network, and this complexity confers elasticity and adaptability to changing environments. Treg cells swarm to areas of immune interrogation and interact with APCs in lymphoid tissues, as do naïve Tconv cells, but they can just as easily migrate in tissues together with T effector cell populations, responding to the same cues; moreover, besides adapting to the environment and downregulating the immune response, Tregs provide molecules which sustain tissue cells, actively participating to tissue protection and regeneration. One prominent site of Treg activity is the intestine, where these cells modulate immune interactions with the microbiota and help maintain tolerance. Changes in microbiota composition in present-day populations, with a prominent representation of proinflammatory microorganisms and a significant reduction of biodiversity, have been shown to underlie the steep increase of autoimmune and allergic disorders observed in western countries, raising the level of inflammation to a point where Treg cells are unable to contrast the fire. It is also possible that subtle defects in Treg function may be unmasked by excessive inflammation, and this may explain why only some individuals succumb to autoimmunity.

Many themes still need to be investigated. For instance, in recent years the role of circadian factors on immune cell function has emerged as a factor which also impacts the development of autoimmunity. Indeed, immune responses have a rhythmicity regulated by intrinsic timers (225, 226), and in animal models susceptibility to sepsis or to EAE induction are significantly and intriguingly dependent on the time of the day (227–230). Only few studies have addressed the issue of circadian rhythms on the functions of Treg cells (231–234), all suggesting that also Treg cells are under circadian control. Also the recently identified resolvins, molecules which mediate resolution of inflammation (235), are a fascinating area of investigation and have been shown to induce Treg cell polarization in humans (236) flipping the balance of immune reactions toward immune regulation and away from inflammation. Additionally, as mentioned, T cell identity is maintained not only by transcriptional programs, and post-transcriptional processes are in place to rapidly tune cell fate decisions in response to changing environments. Non-coding RNAs are RNA transcripts which do not code for protein products but instead regulate gene expression by driving post-transcriptional repression through pairing with mRNA. They include microRNA, small interfering RNA (siRNA), long non-coding RNA, and the recently discovered circular RNA (237–239). The finding that miRNA disruption selectively in Tregs leads to fatal systemic autoimmunity similar to the disease occurring in FoxP3 deficient mice indicates that non-coding mRNAs are central for maintaining Treg cell identity and function (25, 26). Indeed, studies on miRNA and on their role in Treg cell biology have identified a miRNA Treg signature which seems to be conserved across species (240, 241). miRNA are also involved in the pathogenesis of autoimmune diseases, including MS, mainly through the modulation of the Treg/Th17 balance (242–245), and are interestingly found in the extracellular vesicles released by activated lymphocytes. Moreover, they may directly participate to Treg-mediated immune suppression (246).

These areas of investigation may hold the answer to what goes wrong in CNS autoimmunity, although given the complexity of the human meta-organism and of its immune network we have many more than one question, closer to one hundred thousand.

## Author Contributions

GB and MS wrote the manuscript. FG and GG revised the literature. MS and GB conceived and edited the figures. All authors contributed to manuscript revision.

### Conflict of Interest

The authors declare that the research was conducted in the absence of any commercial or financial relationships that could be construed as a potential conflict of interest.
